# Editorial: Vascular- and immuno-metabolism as drivers of cardiovascular disease: insights obtained from omics approaches

**DOI:** 10.3389/fcell.2025.1559828

**Published:** 2025-01-29

**Authors:** Emiel P. C. van der Vorst, Jeffrey Kroon, Raquel Guillamat-Prats, Yvonne Döring

**Affiliations:** ^1^ Department of Internal Medicine I – Cardiology, Uniklinik RWTH Aachen, RWTH Aachen University, Aachen, Germany; ^2^ Institute for Molecular Cardiovascular Research (IMCAR), Uniklinik RWTH Aachen, RWTH Aachen University, Aachen, Germany; ^3^ Aachen-Maastricht Institute for Cardio-Renal Disease (AMICARE), Uniklinik RWTH Aachen, RWTH Aachen University, Aachen, Germany; ^4^ Institute for Cardiovascular Prevention (IPEK), Ludwig-Maximilians-Universität, Munich, Germany; ^5^ Department of Experimental Vascular Medicine, Amsterdam Cardiovascular Sciences, Amsterdam UMC Location University of Amsterdam, Amsterdam, Netherlands; ^6^ Amsterdam Cardiovascular Sciences, Atherosclerosis and Ischemic Syndromes, Amsterdam, Netherlands; ^7^ Laboratory of Angiogenesis and Vascular Metabolism, VIB-KU Leuven Center for Cancer Biology, Leuven, Belgium; ^8^ Laboratory of Angiogenesis and Vascular Metabolism, Department of Oncology, KU Leuven and Leuven Cancer Institute (LKI), Leuven, Belgium; ^9^ Lung Immunity Translational Research Group in Respiratory Diseases, Germans Trias i Pujol Research Institute (IGTP), Badalona, Spain; ^10^ DZHK (German Center for Cardiovascular Research), Partner Site Munich Heart Alliance, Munich, Germany; ^11^ Swiss Cardiovascular Center, Division of Angiology, Inselspital, Bern University Hospital, University of Bern, Bern, Switzerland; ^12^ Department for BioMedical Research (DBMR) Bern University Hospital, University of Bern, Bern, Switzerland

**Keywords:** cardiovascular disease, metabolism, inflammation, immune system, atherosclerosis, omics

Despite previous achievements in the management of cardiovascular disease (CVD), and the fact that the mortality rate from CVD has declined over the last 50 years, atherosclerosis, the chronic condition responsible for the occurrence of a myocardial infarction (MI) and stroke, remains one of the primary causes of global morbidity and mortality. Due to the rising aging population in combination with an increase in cardiometabolic risk factors, primarily driven by the obesity epidemic, the number of individuals affected by CVD is still rising. Therefore, it is of utter importance to develop new strategies aimed at reducing CVD risk and elucidate the molecular mechanisms and important players of atherosclerosis.

Over the past years, it became increasingly clear that atherosclerosis is a multifactorial disease that is not only driven by lipids but also by vascular damage and inflammation (Ajoolabady et al., 2024; Doring et al., 2024; Kong et al., 2022). Compelling evidence that inflammation play a crucial role in atherosclerotic CVD was provided by CANTOS, performed in 2017. Here it was shown that a monoclonal antibody targeting interleukin-1b, termed Canakinumab, effectively reduced CVD risk and mortality, especially in patients characterized with residual inflammation. This effect was independent of lipid-level lowering (Ridker et al., 2017). In late 2019, the inflammation hypothesis of atherosclerosis was confirmed in COLCOT, using the anti-inflammatory agent colchicine in patients with recent MI (Nidorf et al., 2020). A follow-up randomized clinical trial in 2020 applying colchicine involving patients with chronic coronary disease (LoDoCo), also showed significant risk reduction (Tardif et al., 2019). These landmark studies set the stage for identifying drug targets that block atherosclerosis-specific inflammatory pathways as a highly promising strategy to reduce cardiovascular risk.

It is now undisputed that cellular metabolism is important in fueling many pro-atherosclerotic processes in the plethora of cells involved in the disease progression, ranging from endothelial to smooth muscle cells, neutrophils, T and B-lymphocytes and monocytes (Bories and Leitinger, 2017; Domingo et al., 2024; Zhao et al., 2023). Advancing omics technologies provide unprecedented insights into cellular mechanisms, offering a comprehensive and unbiased view of metabolic and immune functions (de Winther et al., 2023; Zhang and Schmidlin, 2024). The articles in this Research Topic provide crucial insights into the role of both vascular- and immuno-metabolism, as important players and drivers of CVD, which is of utmost importance to be able to offer new therapeutic approaches to combat CVD progression.

One of the main challenges of multi-omics approaches is to obtain material for various omics techniques from the same cell population. In their research article, Del Barrio Calvo and Bindila describe a phenotyping approach in which simultaneous extraction of lipids, metabolites and RNA from single cell populations is employed, enabling multi-omic molecular profiling of very low cell numbers. Furthermore, they phenotype MyD88-knockout macrophages as proof of principle and demonstrating the potency of their approach.

Another original research manuscript by Ma et al. deploys bioinformatic analysis and machine learning approaches to evaluate shared pathogenic mechanisms between atherosclerosis and ankylosing spondylitis. They identified ST8 alpha-N-acetyl-neuraminide alpha-2,8-sialyltransferase 4 (ST8SIA4), a polysialyltransferase located in the Golgi apparatus as a key diagnostic marker in the progression of both atherosclerosis as well as ankylosing spondylitis, revealing a component of the common pathological mechanism.

Moreover, Rauterberg et al. investigates the impact of Proprotein convertase subtilisin/kexin type 9 (PCSK9) on the heart function after MI, one of the main clinical outcomes due to atherosclerosis development, showing that the lack of *Pcsk9* in mice improves survival post-MI. Interestingly, Alirocumab (PCSK9 inhibitor) treatment did not replicate these beneficial effects in mice, highlighting that there seems to be important mechanistic differences and differential outcomes between PCSK9 pharmacological inhibition and genetic deficiency.

In the context of MI, Peletier et al. summarizes the current state-of-the-art of cardiovascular 3D models in the context of myocardial ischemia-reperfusion injury (IRI). This elaborate review particularly focusses on the key aspect of cell-cell communication and the potential of multi-omics approaches in these models to enhance our understanding of IRI.

Besides these original research articles, the Research Topic also includes several comprehensive reviews. Indeed, Pi et al. provide a detailed overview of the evolution of atherosclerosis and the involvement of innate and adaptive immune cells in this pathology. Particularly, omics studies, especially single-cell RNA-sequencing studies are discussed to highlight the large degree of cellular heterogeneity within the different immune subsets. These insights are further supported by a review by Annink et al. which also highlights the importance of inflammation and innate and adaptive immune cells in atherosclerosis, emphasizing various approaches that are being pursued in order to identify novel therapeutic targets in this context. Another review focusses specifically on type 2 innate lymphoid cells (ILC2s), which have recently emerged as major regulators of the pathogenesis of various cardiometabolic diseases (Kral et al., 2023). Kral et al. provide a comprehensive overview of the current understanding of ILC2s in inflammation and metabolic disorders. In this review, particularly recent omics studies are discussed that provided crucial insights into the molecular and cellular characteristics of ILC2s, which thereby enhance our understanding of the diversity of this cell type and their involvement in metabolic diseases. Moreover, a review by Dai et al. describes the metabolic cellular changes in macrophages, neutrophils, vascular endothelial cells, vascular smooth muscle cells, and lymphocytes in the context of atherosclerosis and comorbidities. An elaborate understanding of such changes is crucial as it could be shown that various diseases can impact the cellular metabolism, while *vice versa* an altered cellular metabolism can also severely impact disease development.

Another key player in atherosclerosis development is the NOD-like receptor protein 3 (NLRP3) inflammasome, which has been studied extensively already in this context. The systematic review by Miao et al. provide a valuable overview regarding the NLRP3 inflammasome research field over the last decade in the context of CVD. Their analysis reveals leading contributors to the field of NLRP3 research and highlights main pathogenic mechanisms of the NLRP3 inflammasome, like oxidative stress, pyroptosis, and inflammation.

In conclusion, this Research Topic aims to provide a series of articles covering all aspects of how vascular- and immuno-metabolism impact CVD and how metabolic modulation could be used to alter disease progression and thereby contribute to improved diagnostic and therapeutic treatment options in the future.
